# Workforce availability on the intraprocedural stage of endoscopy procedures: a single-center time and motion preliminary efficiency study

**DOI:** 10.1016/j.igie.2023.03.002

**Published:** 2023-04-19

**Authors:** Marco Bassi, Pasquale Apolito, Raffaele Aspide, Annalisa Cappello, Davide Allegri, Cristiano Fabbri, Stefania Ghersi, Giuseppe Indelicato, Carmelo Cascone, Mauro Tiacci, Paolo Tubertini, Pierfrancesco Ghedini, Stefano Guicciardi, Vincenzo Cennamo

**Affiliations:** 1 Gastroenterology and Interventional Endoscopy Unit, Local Health Authority of Bologna, Bologna, Italy; 3 Department of Clinical Governance and Quality, Local Health Authority of Bologna, Bologna, Italy; 4 Health Management, Local Health Authority of Bologna, Bologna, Italy; 5 Health Directorate, Local Health Authority of Bologna, Bologna, Italy; 7 Communication and Informatics Technology Unit; 2 Anesthesia and Intensive Care Unit, IRCCS Istituto delle Scienze Neurologiche di Bologna, Bologna, Italy; 6 Enterprise Information Systems for Integrated Care and Research Data Management, IRCCS Azienda Ospedaliero-Universitaria di Bologna, Bologna, Italy; 8 Department of Biomedical and Neuromotor Sciences, University of Bologna, Bologna, Italy

## Abstract

**Background and Aims:**

An increased demand for endoscopy facilities has stimulated studies on efficiency. This study used a time and motion method to evaluate the effective use of healthcare resources in the intraprocedural phase of endoscopy procedures in a GI department of a tertiary care referral hospital and to identify potential recruitable resources.

**Methods:**

Consecutive EGD, colonoscopy, and ERCP were prospectively observed during the study period. For all procedures, total duration in the endoscopy room (TDE) and endoscopy procedure time (PT) were mapped. In the EGD and colonoscopy groups, the activity and “available time” of a second allocated nurse were evaluated. In the ERCP group, endoscopy activity and off-work time of endoscopy staff were evaluated.

**Results:**

Eighty-seven EGDs, 77 colonoscopies, and 55 ERCPs were analyzed. The mean TDE was 27.06 minutes for EGD, 39.32 minutes for colonoscopy, and 67.12 minutes for ERCP. The average PT was 11.31 minutes for EGD, 23.21 minutes for colonoscopy, and 38 minutes for ERCP. The second nurse executed the task in 66.6% of EGDs and 58.4% of colonoscopies, with a mean available time with respect to the mean TDE of 19.52 minutes for EGD and 24.33 minutes for colonoscopy. In the ERCP group, 1 endoscopist and 1 nurse were off-work for an average time of 28.34 minutes per procedure and in the first 10 minutes after patient entry into the room in 80% of cases.

**Conclusions:**

Even with the limitations of a single-center study, our time and motion study showed potentially recruitable healthcare resources during the procedural phase of endoscopic procedures.

Increasing demand for healthcare services, multifactorial cost increases, and reduced staffing levels make it essential to develop processes to increase efficiency. Endoscopic staff resources and their assignments, based on country regulations and hospital internal organization, significantly affect the costs of endoscopy centers.^1^^,^^2^ Therefore, staff optimization is a crucial issue because of decreasing reimbursements for procedures and the shortage of specialized personnel, such as registered nurses.^3^ Moreover, in the coronavirus disease 2019 pandemic era, staff was periodically asked to work additional shifts because of personnel shortages and reallocation to coronavirus disease 2019 departments.^4^

Time and motion study (TMS), widespread in the industrial setting, is the independent and continuous observation of a specific task to analyze the time spent performing a series of clinical or nonclinical activities that are recorded using 3 different methods: an external observer, self-reporting, or automatically by computerized systems.^5^ Modified observational TMS approaches to analyze how healthcare workers spend their time during their working shifts, including quantitative and qualitative evaluation, have been applied.^6^, ^7^, ^8^, ^9^ TMS was used in endoscopy efficiency studies to assess patient flow metrics as time intervals of each endoscopic procedure or such specific features as procedure duration or its start delays.^10^, ^11^, ^12^, ^13^ So far, data regarding the use of the human resources during an endoscopy procedure are lacking.

The first aim of our study was to assess the available time and probabilistic availability of the nurse dedicated to the endoscopic task during EGD and colonoscopy, with respect to the total duration of the procedure, applying the TMS method. The secondary aim was to evaluate the available time and probabilistic availability of the endoscopy team (including the endoscopist and the nurse dedicated to the endoscopic task) in ERCP with anesthesia physician–assisted sedation, with respect to the total duration of the procedure, applying the TMS method.

## Methods

### Setting and patient population

This single-center, prospective observational study was conducted at Maggiore Hospital in Bologna (Italy), a tertiary care referral hospital in northern Italy, from November 2020 to January 2021. The GI endoscopy unit, where the present study was carried out, consists of 3 endoscopy suites (ESs), equipped for nonoperating room anesthesia, and 1 recovery and preparation room (RR) with 8 beds. Two ESs are open full time and dedicated to EGD and colonoscopy. The third ES is equipped also with fluoroscopy and is opened as needed for ERCP, GI stent placement, and third-space endoscopy procedures.

Endoscopy procedure volume is over 5500 cases per year, consisting of a wide case-mix of GI procedures including a combination of inpatient procedures and elective outpatient procedures. The unit is operated from 8:00 AM to 8:00 PM for elective procedures (Monday to Friday); urgent procedures are processed 24 hours a day, 7 days a week.

Eight gastroenterologists use the endoscopy unit, alternating on a weekly rotation; 3 of them are also experts in ERCP. Up to 6 full-time endoscopy registered nurses work in each shift in the unit: 2 nurses per ES and 2 are involved in the management of the RR. Each procedure involves an endoscopist; a first nurse (A), who takes care of the patient from and to the RR, monitoring vital signs and sedation; and a second nurse (B), involved in endoscopic tasks. For additional procedures (ie, urgent endoscopic procedures), the nurse from the RR alerts through intercom the working staff in each room.

The scheduled time for each procedure included procedure duration, anesthesia time for ERCP, and room turnover time (RTT). A daily procedure timetable is settled according to predefined allotted times: 30 minutes for EGD, 45 minutes for colonoscopy, and 60 minutes for ERCP. EGD and colonoscopy are performed with the endoscopist directing mild sedation, administering a combination of a benzodiazepine (midazolam) and an opioid (meperidine) under monitoring of vital signs. Equipment preparation (ie, check for endoscope functioning) is performed before the procedure by nurse A. When intraprocedural tasks (ie, polypectomy, biopsy sampling) are requested, nurse B brings the accessory from the dedicated trolley.

ERCP is performed with the patient under general anesthesia with a dedicated anesthesiologist. In this setting, nurse A prepares equipment for the procedure and provides support to the anesthesiologist, and nurse B assists with technical endoscopy–related tasks. During anesthesia induction and awakening, the endoscopist and nurse B do not have specific tasks.

Patient workflow is carried out as follows. Inpatients and outpatients are first taken into the RR where medical history, written consent, and intravenous access placement are performed. Patients are then moved to the ES. When the procedure ends, the endoscopist sees to the reporting, and patients are transferred to the RR and then discharged after reaching the target criteria.

### TMS methods

The present study focused the TMS on the intraprocedural stage, which is the set of activities between patient entry and exit from the ES. We used double-checked TMS methodology combining data produced by an external observer (a gastroenterologist fellow) with 1:1 continuous observation and recording data produced by each staff member involved using the dedicated software, Sportmodeling (Nextworks srl, Pisa, Italy).

Sportmodeling is an experimental software designed to track the quantitative data regarding patient entry and exit from the ES and procedure duration and activity of each physician and nurse through self-reported 1-click time stamps. The software records operators’ log-in and log-out times. Sportmodeling is an electronic time capture tool developed to support data collection for TMS studies that is optimized for touch-enabled Android tablets, installed in each ES and in the RR. At the same time, the external observer noted the type of task performed, thus avoiding the staff measurement being biased by spending additional time in reporting it.

### Data collection

Procedures included in the analysis were all consecutive EGDs, colonoscopies, and ERCPs performed in the period under consideration. Data were subsequently extrapolated from electronic database records of Sportmodeling and external observer notes. The following events were extracted: time of entry and exit of the patient from the ES, defined as the total duration in the endoscopy room (TDE); time of insertion and removal of the endoscope, defined as the procedure time (PT); time between patient entrance and endoscope insertion, defined as the preoperative time; time between endoscope removal and patient discharge from the suite, defined as the postoperative time; nurse B request rate, duration, and tasks; nurse B activity in the cumulative distribution; nurse B availability in the cumulative distribution; endoscopic staff off-work time; endoscopy staff availability in the cumulative distribution; and time between patient exit and entry of subsequent patient into the ES, defined as the RTT.

### Statistical analysis

Time variables are expressed as descriptive statistics in minutes averaged with the confidence interval (CI). The time of examination was standardized to obtain a log normal distribution for all procedures studied. The Z score was applied for probability evaluation. Data were analyzed using SPSS (version 20.0, released 2011; IBM SPSS Statistics for Windows, Armonk, NY, USA).

### Ethics approval and consent to participate

The study was performed in accordance with the ethical standards as laid down in the 1964 Declaration of Helsinki and its later amendments or comparable ethical standards. Data collection was completely anonymous with respect to both patients (cumulative data) and participating physicians and nurses. Because of the regulations of local ethics, the ethical review board was not required.

## Results

### EGD and colonoscopy

Of 164 analyzed procedures, 87 were EGDs and 77 colonoscopies. The TDE average was 27.06 minutes (95% CI, 24.47-29.24) for EGD and 39.32 minutes (95% CI, 35.51-43.13) for colonoscopy. In the ES where EGD and colonoscopy were performed, the average RTT was 8.34 minutes (95% CI, 6.31-10.37). The average PT was 11.31 minutes (95% CI, 9.59-12.03) for EGD and 23.21 minutes (95% CI, 22.39-24.03) for colonoscopy (Table 1). The mean preoperative time room for EGD and colonoscopy were 8.18 and 8.33 minutes, respectively. The mean postoperative time was 7.17 minutes for EGD and 7.38 minutes for colonoscopy.

**Table 1:** EGD and colonoscopy metrics

	No. of cases	Total duration in endoscopy room		Procedure time	
		Average time (min)	95% Confidence interval	Average time (min)	95% Confidence interval
EGD	87	27.06	24.47-29.24	11.31	10.58-12.04
Colonoscopy	77	39.32	35.51-43.13	23.21	22.39-24.03

Nurse B was requested to perform a task in 66.6% of EGDs (58/87 procedures) and 58.4% of colonoscopies (45/77 procedures). The average nurse B task duration was 7.14 minutes for EGD (95% CI, 5.39-8.49) and 14.59 minutes for colonoscopy (95% CI, 10.39-19.19). All results and specific tasks are shown in Table 2. When nurse B was requested for a task, the task distribution according to TDE and PT was evaluated.

**Table 2:** Nurse B tasks for EGD and colonoscopy

Nurse B task	No. of procedures (%) with nurse B requested (n = 87)	Average time (min) (95% confidence interval)
*EGD*		
All tasks	58 (66.6)	7.14 (5.39-8.49)
PEG placement	2 (2.2)	8.30 (7.55-9.05)
Nasogastric tube placement	1 (1.1)	9.02 (—)
Esophageal varices banding	3 (3.4)	16 (7.32 -8.28)
Patient agitation	2 (2.2)	6.30 (5.49-7.11)
Biopsy sampling	43 (49.4)	4.54 (4.10-5.38)
Pancreatic stent removal	3 (3.4)	7.00 (3.25-10.35)
Esophageal sponge removal	1 (2.2)	26.00 (—)
GI bleeding (hemostasis)	3 (3.4)	23.40 (18.36-28.44)
	No. of procedures (%) with nurse B requested (n = 77)	

*Colonoscopy*		
All tasks	45 (58.4)	14.59 (10.39-19.19)
Biopsy sampling	14 (18.2)	9.43 (7.52-11.34)
Polypectomy	14 (18.2)	20.40 (12.24-28.56)
Abdominal manipulation	5 (6.5)	2.48 (1.12-4.24)
Abdominal manipulation and patient agitation	1 (1.3)	9.06 (—)
Abdominal manipulation and polypectomy	3 (3.9)	18.40 (5.34-31.46)
Biopsy sampling and polypectomy	1 (1.3)	30.11 (—)
Conversion to 2-operator colonoscopy	2 (2.6)	27.30 (24.06-30.54)
Stabilization of the endoscope	1 (1.3)	2.14 (—)
Abdominal manipulation and stabilization of the endoscope	3 (3.9)	3.40 (2.16-5.04)
Conversion to endoscopic submucosal dissection	1 (1.3)	70.00 (—)

—, 95% CI not calculable.

**Figure 1:**
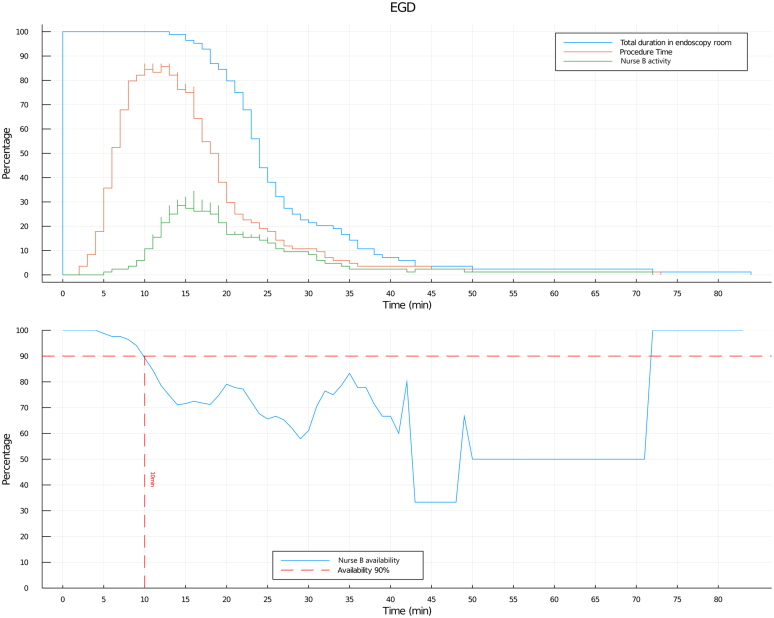
Total duration in the endoscopy room (TDE), procedure time (PT), and nurse B activity and availability for EGD. (*Top*) Log normal distribution of TDE, PT, and nurse B activity. (*Bottom*) Nurse B availability (calculated as inverse of nurse B activity).

**Figure 2:**
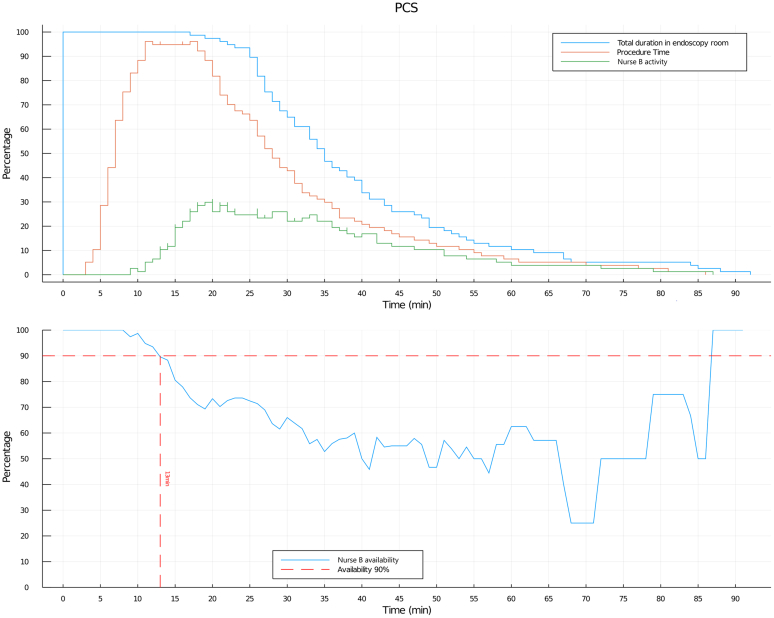
Total duration in the endoscopy room (TDE), procedure time (PT), and nurse B activity and availability for colonoscopy (PCS). (*Top*) Log normal distribution of TDE, PT, and nurse B activity. (*Bottom*) Nurse B availability (calculated as inverse of nurse B activity).

Based on the average duration per procedure for all tasks of nurse B (Table 2), subtracting the average TDE, a mean nurse B available time of 19.52 minutes for EGD and 24.33 minutes for colonoscopy, each amounting to 73.3% and 62%, respectively, of the mean TDE, was extracted. Conversely, relying on the average time per procedure for all tasks of nurse B (Table 2), when considering the PT, a mean nurse B available time of 4.17 minutes for EGD and 8.22 minutes for colonoscopy, each amounting to 37.2% and 35.8%, respectively, of mean PT, was obtained. According to the log normal distribution of the activity of nurse B and TDE, nurse B was requested for tasks after an average time of 15.51 minutes for EGD and 19.41 minutes for colonoscopy and was off-work on average for the last 7.28 minutes for EGD and 10 minutes for colonoscopy. Nurse B was off-work in 90% of the cases, in the first 10 minutes of an EGD and in the first 13 minutes of a colonoscopy (Figs. 1 and 2). In procedures where nurse B was requested, applying the Z score, there was a 90% probability of a nurse B request for a task in 27.30 minutes for EGD and in 29 minutes for colonoscopy after patient entry into the ES. Furthermore, analyzing an arbitrary cutoff of 11 minutes, the probability that nurse B was requested in the first 11 minutes was 30.15% for EGD and 11% for colonoscopy.

### ERCP

Fifty-five ERCP procedures were analyzed. The average TDE was 67.12 minutes (95% CI, 60.00-73.42), the average PT average was 38 minutes (95% CI, 32.55-43.35), and the average RTT was 14.36 minutes (95% CI, 11.02-18.10). The preoperative time was an average of 15.04 minutes (95% CI, 13.49-16.19) and the postoperative time an average of 13.53 minutes (95% CI, 12.35 -15.11).

**Figure 3:**
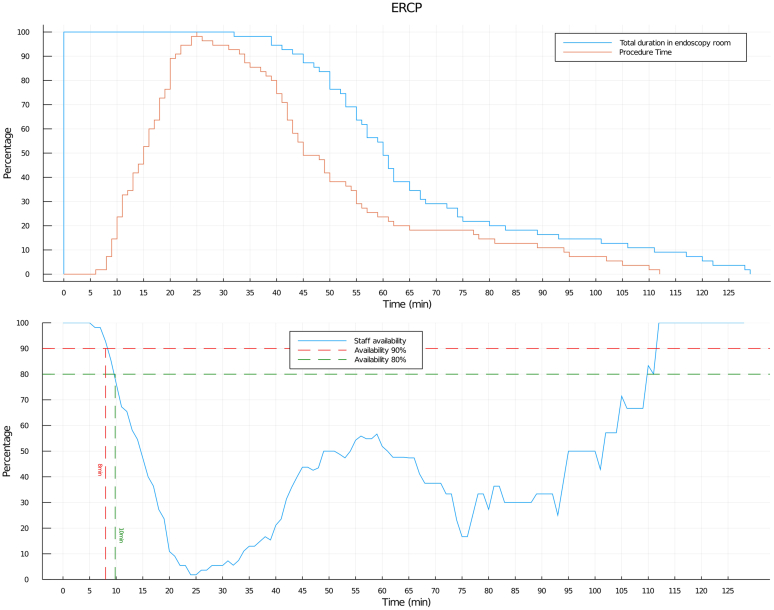
Total duration in the endoscopy room (TDE), procedure time (PT), and staff availability for ERCP. (*Top*) Log normal distribution of TDE and PT. (*Bottom*) Endoscopy staff availability (calculated as inverse of PT log normal distribution).

Adding up data for the 2 steps provided an estimate that an endoscopist and nurse B were off-work for an average total time of 28.34 minutes (95% CI, 25.55-30.06) (Table 3). Notably, Z score evaluation showed a 50.52% probability for the endoscopy staff to be off-work from a minimum of 20 to a maximum of 32 minutes. Finally, log normal distribution showed that the endoscopist and nurse B were off-work for the first 10 minutes in 80% and for 8 minutes in 90% of analyzed cases (Fig. 3). During the PT timeline, no available time was shown in the time log for nurse B and the endoscopist.

**Table 3:** ERCP metrics

	Average time (min)	95% Confidence interval
Total duration in the endoscopy room	67.12	60-73.42
Procedure time	38	32.55-43.35
Preoperative time	15.04	13.49-16.19
Postoperative time	13.53	12.35-15.11
Endoscopy staff Off-work time	28.34	25.55-30.06

## Discussion

To our knowledge, this is the first TMS assessing the length and features of staff members time spent on an intraprocedural phase in a gastroenterology endoscopy center. Furthermore, the study detailed the duration, different tasks, and timeline distribution of the nurses involved, and, consequently, it estimated staff inactivity time.

From a different point of view, nurse B did not perform any task in 33.4% of EGDs (29/87) and in 41.6% of colonoscopies (32/77). Furthermore, when requested to perform tasks, nurse B was off-work in 73.3% and 62% of the TDE for EGD and colonoscopy, respectively. Notably, intraprocedural inactivity as high as 37.2% and 35.8% for EGD and colonoscopy, respectively, were found for nurse B during the PT.

Based on our organization, which provides a second nurse in all procedures, our findings showed that the workforce was not always involved in the activities. In fact, from patient entry, nurse B was free in 90% of cases analyzed in the first 10 minutes of an EGD and in the first 13 minutes of a colonoscopy, irrespective of procedure length. Additionally, according to the average nurse B task time per procedure, the available time during the PT turned out to be lower for EGD than for colonoscopy.

In ERCP, we found that the endoscopist and nurse B were simultaneously off-work (“endoscopy staff off-work time”) for an average total time of 28.34 minutes of TDE from the patient’s induction of and awakening from anesthesia; conversely, no endoscopy staff member was available during the PT. Furthermore, during ERCP, in the first 10 minutes, the endoscopist and nurse B were simultaneously off-work in 80% of the cases. The first 10 minutes in EGD, colonoscopy, and even in ERCP seem to be a good cutoff at which to consider nurse B available. Notably, our study not only sheds light on the length of inactivity time of operators during the procedure, but it also shows a probabilistic pattern of inactivity of 1 or more operators, simultaneously involved in the same procedure, despite the procedure’s duration.

So far, TMS has been part of a lean management strategy, which increases efficiency by optimization of nonvalue-added tasks of a linear process.^14^ In this perspective, previous TMS findings reported yielding endoscopy workflow metrics and process inefficiencies. Furthermore, they showed that the PT is rarely rate-limiting, whereas the nonprocedural operational flow processes of preprocedure and RR times were instead crucial targets for improvement.^11^^,^^12^^,^^15^ To minimize the RR time, obtaining prior consent, intravenous access placement, and sedation induction by a nonendoscopist have been suggested by other authors.^16^^,^^17^

Parallel processing, a model drawn from the field of surgery, was implemented with 2 rooms running in parallel using the same physician but reallocating personnel during RTT.^18^ In the endoscopy field, “3 rooms for 2 endoscopists'' or “2 rooms per operator” have been proposed as a parallel processing workflow model.^12^^,^^16^, ^17^, ^18^ In particular, the “2 rooms for 1 endoscopist” model seems to increase procedure volume and efficiency but requires dedicated additional staff for the second room (up to 4.5 full-time equivalents), resulting in increased costs.^16^ Furthermore, Zamir and Rex^17^ suggested that another prerequisite for applying this model would be a high procedure volume per each endoscopist involved, which is in contrast to the wide variation of procedure volume (up to 3-fold variation) between endoscopists encountered in real practice. However, the strongest predictor of procedure volume was not the actual PT but RTT.^17^

In our study we reported a significant amount of RTT for all the procedures. Notably, the RTT for EGD and colonoscopy resulted in approximately 8 minutes, which is in line with the experience of Almeida et al.^12^ Therefore, RTT would be useful for future interventions, such as staggering the PT schedule between 2 parallel working ESs, to potentially increase personnel available for recruitment or future analysis of factors affecting ES turnover. Our data support a potential reallocation of tasks by using the same staff during the intraprocedural phase, including parallel workflow processing.

In summary, according to the average task duration per procedure per nurse B and to inactivity patterns of staff members, we hypothesize a potentially new workflow model: the use of parallel processing “on demand” in relation to staff availability. The actual availability predicted must be identified by a management system that continuously shows in real time daily workflow changes and workforce availability, thereby allowing the on-demand reallocation of personnel for 2 rooms running in parallel or the overcoming of any emerging needs. This kind of information and relative action could most likely be managed by an event-driven organization model where specific events should trigger a workflow or decision in real time through dynamic activation with reallocation of resources.^19^ A practical example of this approach can be seen in basketball, where players are able to perform a real-time reactive implementation of strategies. To tackle unplanned events, their decision-making is based on a set of schemes learned during training.^20^ Similarly, in the endoscopy center scenario, there are limited procedural events that can occur, and operators should be trained to face them.

**Figure 4:**
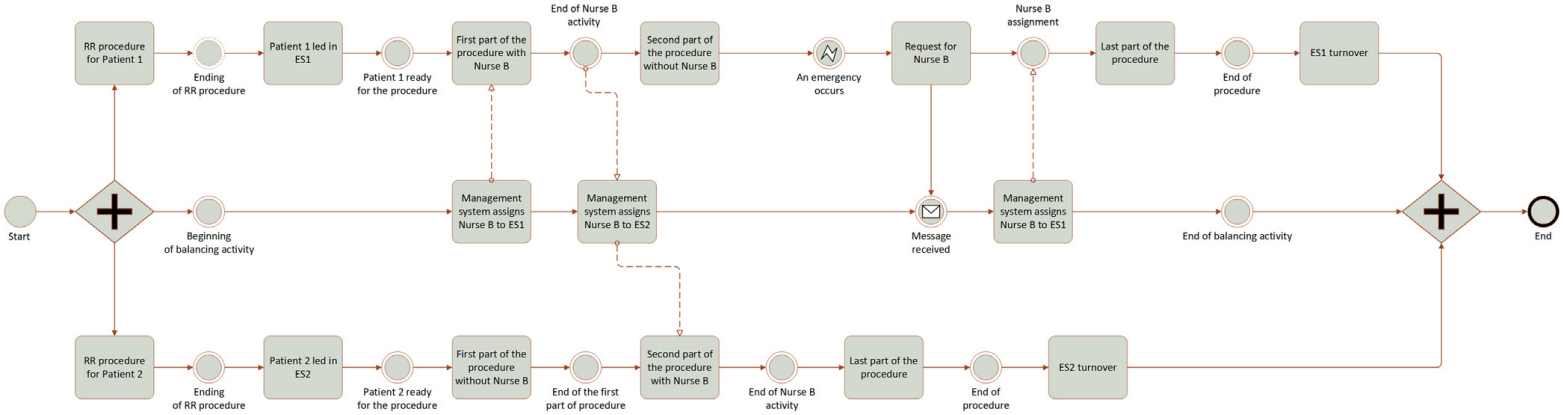
Example of event-driven nurse allocation. *RR*, Recovery and preparation room; *ES*, endoscopy suite.

An example of this new type of workflow model was developed through the business process model and notation and is shown in Figure 4.^21^ It depicts a process where 2 different procedures are performed in parallel with a management system that works as a “load balancer” with a dynamic assignment of nurse B based on probability of use and emergency occurrence. The process starts with the RR procedures for both patients 1 and 2. After the 2 patients are taken to their ESs, the procedures can begin. The management system assigns nurse B to ES1 because this type of procedure is more likely to require its use in the first part. Once nurse B completes the task, the management system moves the nurse to ES2 because it is now more likely that procedure 2 will need additional staff. Suddenly, an emergency occurs in ES1, which is reported to the management system, which moves nurse B back to ES1. Subsequently, the 2 procedures end. As shown, the proposed approach allows nurse B to be assigned dynamically, exploiting a prediction of the downtime associated with the specific procedure.

TMS collection methods are exposed to potential intrinsic limitations. On the one hand, self-report–based TMS collections are considered untrustworthy, because there seems to be an overestimation of efficiency metrics and underestimation of nonproductive clinical time.^5^^,^^22^ On the other hand, “pure” external observer-based TMSs are affected by apparent increased performances of the subject studied, a phenomenon known as the Hawthorne effect.^5^^,^^23^ The strength of our study is in the use of a hybrid TMS approach, a combination of self-report–based and external observer data collection, with the aim to reduce the bias of each TMS single method. This method has provided information on the effective use of resources in the most consistent, complete, and accurate form. So far, parallel room implementation has been based on the use of additional staff, not on the reallocation of off-work staff during the course of the procedure, as theoretically suggested by our study.^16^

Some limitations of this study are noteworthy. First, the study represents the experience of a single endoscopy unit in a referral hospital, with 2 nurses and 1 endoscopist allotted per room; additionally, the RR is not guaranteed worldwide. Therefore, these observations could not be extended to other centers. Second, although we showed an intraprocedural, predictable availability of personnel, this is not able to be standardized because it strictly depends on tasks that arise because of unplanned event occurrence, and so it is not suitable for lean management efficiency interventions. Third, statistical power analysis was not performed before data collection, because data collection occurred only over a limited period. Furthermore, this period was additionally affected by seasonal and pandemic variations in hospital admissions.

In conclusion, in an endoscopy department, predictable resources, because of their inactivity during a procedure’s timeline, could potentially be reallocated to perform other tasks during the course of the procedure, without potentially any additional workforce cost. However, we are planning to validate this evidence in a future randomized control trial, even multicentric, to compare a standard workflow model with the aforementioned “parallel” model to evaluate performance but also any potential safety issue. This is of particular interest in an era where economic aspects of medical care are becoming increasingly important. However, additional and prospective data will be needed to evaluate the feasibility of the application of this model in real practice.
